# Transforming and evaluating electronic health record disease phenotyping algorithms using the OMOP common data model: a case study in heart failure

**DOI:** 10.1093/jamiaopen/ooab001

**Published:** 2021-02-04

**Authors:** Vaclav Papez, Maxim Moinat, Stefan Payralbe, Folkert W Asselbergs, R Thomas Lumbers, Harry Hemingway, Richard Dobson, Spiros Denaxas

**Affiliations:** 1 Institute of Health Informatics, University College London, London, UK; 2 Health Data Research UK, London, UK; 3 The Hyve, Utrecht, Netherlands; 4 Department of Cardiology, Division Heart & Lungs, University Medical Center Utrecht, Utrecht University, Utrecht, the Netherlands; 5 Department of Biostatistics and Health Informatics, Institute of Psychiatry, Psychology and Neuroscience (IoPPN), King’s College London, London, UK; 6 The Alan Turing Institute, London, UK

**Keywords:** heart failure, EHR, phenotyping, OMOP, algorithms

## Abstract

**Objective:**

The aim of the study was to transform a resource of linked electronic health records (EHR) to the OMOP common data model (CDM) and evaluate the process in terms of syntactic and semantic consistency and quality when implementing disease and risk factor phenotyping algorithms.

**Materials and Methods:**

Using heart failure (HF) as an exemplar, we represented three national EHR sources (Clinical Practice Research Datalink, Hospital Episode Statistics Admitted Patient Care, Office for National Statistics) into the OMOP CDM 5.2. We compared the original and CDM HF patient population by calculating and presenting descriptive statistics of demographics, related comorbidities, and relevant clinical biomarkers.

**Results:**

We identified a cohort of 502 536 patients with the incident and prevalent HF and converted 1 099 195 384 rows of data from 216 581 914 encounters across three EHR sources to the OMOP CDM. The largest percentage (65%) of unmapped events was related to medication prescriptions in primary care. The average coverage of source vocabularies was >98% with the exception of laboratory tests recorded in primary care. The raw and transformed data were similar in terms of demographics and comorbidities with the largest difference observed being 3.78% in the prevalence of chronic obstructive pulmonary disease (COPD).

**Conclusion:**

Our study demonstrated that the OMOP CDM can successfully be applied to convert EHR linked across multiple healthcare settings and represent phenotyping algorithms spanning multiple sources. Similar to previous research, challenges mapping primary care prescriptions and laboratory measurements still persist and require further work. The use of OMOP CDM in national UK EHR is a valuable research tool that can enable large-scale reproducible observational research.

## Lay Summary

Doing research on health data from different sources is challenging because often information is stored in different ways and using varying formats. As a result, researchers need to spend substantial amounts of time to harmonize such data before analyses can be done. A potential way to address this challenge is to convert data to a common data specification shared across all sources. In our study, we converted a large data set of electronic health data to the OMOP common data model (CDM). We evaluated the conversion by examining the original and converted data and calculating statistical measurements on various important clinical features. We observed a good agreement between the datasets and converted the majority of information in the data with very low information loss. Our research demonstrates how CDM approaches can be used to convert electronic health data from primary care and hospitalizations to a common format that can enable research to improve human health and healthcare.

## BACKGROUND AND SIGNIFICANCE

The combination of electronic health record data with large biobank cohort studies (eg UK Biobank,^1^ eMERGE^2^) has scaled the breadth and depth of genetic discoveries to identify hundreds of thousands of novel associations between variants and phenotypes (and endotypes) derived from EHR through analyses such as phenome-wide association studies (PheWAS).^3^ EHR data however pose significant challenges as they are collected as part of clinical care or for administrative purposes and not research. Information contained in EHRs is often stored in bespoke formats, using a range of controlled clinical terminologies and is of variable data quality.^4^ These challenges are amplified when using data from multiple EHR sources that lack a common data definition standard, and a significant amount of preprocessing is required to harmonize and transform data into a data set that can then be integrated with genetic discovery pipelines. Common data models (CDMs) can potentially address these challenges by harmonizing EHR data across multiple sources under a standardized format.

CDMs, such as the OMOP CDM,^5^ managed by the Observational Health Data Science and Informatics (OHDSI) community, or the PCORNet CDM,^6^ enable researchers to integrate and analyze information contained in disparate observational data sources by mapping data (and the associated vocabularies used to record that data) into a common format with a robust specification. The OMOP CDM encapsulates definitions for patients, healthcare providers, clinical encounters, and healthcare related concepts (such as a diagnosis or a laboratory measurement) recorded during those encounters. OMOP CDM has been widely used to transform large observational EHR databases. In the United States and elsewhere, researchers have converted EHR and claims data to the OMOP CDM to enable federated analyses of disparate sources of information.^7^^,^^8^ In the United Kingdom, the Clinical Research Practice Research Datalink (CPRD)^9^ and The Health Improvement Network (THIN)^10^ have been converted to the OMOP CDM. In both of these cases, extensive transformations were performed to map bespoke data provider formats and UK-specific clinical terminologies and researchers evaluated the quality of the CDM in terms of replicating existing epidemiological analyses performed in the raw data sources.

Previous studies however have been mostly limited to disease phenotypes from single sources (eg only primary care EHR) and the ability of the CDM to adequately transform EHR spanning multiple sources (eg primary care and hospitalizations and mortality) has not been fully evaluated. Linked EHR enable researchers to recreate the longitudinal patient pathway spanning across healthcare settings and to obtain rich longitudinal phenotypes on risk factors and covariates leveraging multiple sources.^11^^,^^12^ The use of multiple data sources however in EHR phenotyping algorithms introduces a higher level of complexity in terms of data volume, variability and consistency,^13^ all of which may be potentially addressed by mapping the source data into the OMOP CDM.

The aim of this study was to evaluate the feasibility of converting and representing phenotyping algorithms which utilize EHR from linked primary care, hospitalization records and a mortality registry from the United Kingdom into the OMOP CDM. We evaluate the conversion process by calculating and comparing descriptive statistics on syntactic (eg percentage of terms mapped from source controlled clinical terminologies) and clinical (eg prevalence of related comorbidities) metrics. We use heart failure (HF) as a case study as it exemplifies the challenges and opportunities of working across EHR sources spanning multiple healthcare settings.

## METHODS

### OMOP common data model

We used version 5.2 of the OMOP CDM which consists of 24 data tables organized in four top-level domains: clinical, derived elements, health system, and health economics. Clinical data tables (*n* = 14) hold core data on patient demographics, clinical events (eg diagnoses, laboratory measurements, medication prescriptions, and surgical procedures), visit occurrences and observation periods. Four clinical tables were not populated since the source EHR did not contain specimen information or free text data. We preprocessed clinical events such as drug exposure periods and stored information as derived elements (*n* = 5). The health system data tables (*n* = 3) provide information on healthcare providers associated with the healthcare events held in the clinical data types. Finally, the health economics data tables (*n* = 2, none were populated) contain cost information and details on enrollment of patients in health benefit plans. More information on the CDM specification can be found on the OHDSI repository^14^

The basic units used to express clinical information across all domain tables are called “concepts” and have a unique identifier within the OMOP CDM (we will denote CDM concepts as Concepts from this point onwards in the manuscript). Concepts can represent broad disease categories (eg “Cardiovascular disease”), detailed clinical elements (eg “Myocardial infarction of the anterolateral wall”), or modifying characteristics and attributes that define Concepts at various levels of detail (eg severity of disease). The CDM contains tables of standardized Concepts which are derived from international standards such as SNOMED-CT, RxNorm, LOINC, and a mapping between Concepts and terms in each controlled clinical terminology are provided, and we utilized the OMOP vocabulary version dated 01/12/2017. An extensive online browser of the OMOP vocabularies is available by using ATHENA^15^

### CALIBER EHR resource

The CALIBER resource^16^^,^^17^ is a library of phenotyping algorithms and methods based on three national EHR sources. The CALIBER resource curates rule-based EHR phenotyping algorithms defining (1) disease status, onset, severity, (2) biomarker measurements (eg neutrophils, blood pressure, body mass index^18–20^), and (3) lifestyle risk factors (eg alcohol consumption, smoking, ethnicity^21–23^). A particular focus of the platform has been reproducibility and portability.^24^ CALIBER phenotypes are curated in an online open-access portal for researchers and clinicians under an open-access license.^25^ CALIBER phenotypes have been used in >100 observational epidemiology studies including recent studies investigating direct and indirect excess deaths due to Coronavirus disease (COVID-19).^26^^,^^27^

The baseline cohort is composed of a national primary care EHR database, the Clinical Practice Research Datalink (CPRD).^28^ CPRD contains longitudinal primary care data (extracted from the Vision and Egton Medical Information Systems clinical information systems) on diagnoses, symptoms, laboratory tests, drug prescriptions, vaccinations, blood tests, and lifestyle risk factors. These are collected during consultations with a primary care physician and are irrespective of disease status and hospitalization. Data are recorded using Read terms V2 (approximately 100 000 terms which are a subset of the International Health Terminology Standards Development Organization SNOMED-CT [Systematized Nomenclature of Medicine Clinical Terms]).^29^ Prescriptions are recorded using Gemscript (a commercial derivative of the NHS Dictionary of Medicines and Devices [DM+D]) (approximately 67 000 terms).^30^ CPRD data have been shown to be representative of age, sex, mortality, and ethnicity and of high diagnostic validity.^22^^,^^31–33^

Hospital Episode Statistics (HES)^34^ contains administrative data on diagnoses and procedures generated during hospital admissions. Diagnoses are recorded using the ICD-10 system and procedures using the Office of Population Censuses and Surveys Classification of Surgical Operations and Procedures, Fourth Revision (approximately 8500 terms, similar to Current Procedural Terminology^35^). Up to 20 primary and secondary discharge diagnoses are recorded per finished consultant episode. The Office for National Statistics (ONS) contains socioeconomic deprivation using the Index of Multiple Deprivation^36^ and physician-certified cause-specific mortality (underlying and up to 14 secondary causes using International Classification of Diseases-Ninth Revision [ICD-9] or ICD-10).

### Study population

We identified HF patients by extracting diagnoses in primary care (CPRD), hospitalizations (HES), and death certificate information (ONS) using a previously defined and validated EHR phenotyping algorithm. Briefly, the diagnosis of HF was based on Read codes for CPRD data and International Classification of Diseases (ICD)‐9 or -10 codes in HES and ONS which are created using a systematic approach described elsewhere in detail.^25^ The algorithm is available online (http://portal.caliberresearch.org/) and described in detail elsewhere.^25–27^

Patient follow-up started in patients diagnosed with incident heart failure from 1st of January 1998 onwards and ended with (1) patients’ survival and patient morbidity as outcome, (2) death in CPRD or ONS, (3) transfer out of GP practice in CPRD, (4) study end date (31 July 2016), (5) last collection date from the practice and end of data collection from HES and ONS, whichever came first. We excluded patients under 18 years of age and with less than a year of follow-up prior to HF diagnosis.

### Transforming CALIBER EHR to OMOP CDM

**Figure 1:**
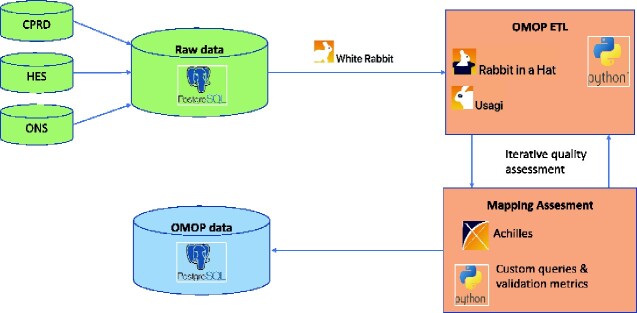
Overview of the transformation process of raw electronic health records linked from three national sources to the OMOP common data model. The main steps of the process are the following: (A) Raw data from primary care (CPRD), hospitalizations (HES) and mortality (ONS) are migrated and loaded in a Postgres relational database system. (B) White Rabbit summary reports are generated and inform the design of the ETL pipeline; (C) Working with experts of the source data, syntactic mappings are generated using the “Rabbit In a Hat” tool and mappings between vocabularies are created using the “Usagi” tool; (D) In an iterative manner, raw data are passed through the ETL pipeline, mapping quality is assessed using the “Achilles” tool and bespoke queries/validation metrics and the ETL mappings are refined; (E) the final data set is stored in a Postgres relational database and is queried to produce datasets for statistical analyses. CPRD, clinical practice research datalink; HES, Hospital Episode Statistics; ONS, Office for National Statistics; OMOP, Observational Medical Outcomes Partnership (OMOP); CDM, common data model; ETL, extract transform load.

We created a bespoke Extract Transform Load (ETL) process which was composed of two parts: syntactic and semantic mapping (Figure 1). The syntactic ETL process mapped data fields in CALIBER to CDM fields while the semantic mapping process created mappings between controlled clinical terminologies and CDM Concepts or mapped data source specific fields (eg smoking-related information such as packs per year and smoking status recorded during primary care consultations) to CDM concepts. Using the OHDSI WhiteRabbit and Rabbit in a Hat tools, we documented the mappings between tables (eg, the *patient* table in CPRD mapped to the *person* table in the CDM) and between columns (eg the *patid* column which contains a unique pseudoidentifier in CPRD mapped to the *person_id* column in the CDM).

We used existing OHDSI vocabulary mappings to map the controlled clinical terminologies used in the source EHR data sources (ie Read V2, OPCS-4, ICD-10) to OHDSI standard Concepts which are based on SNOMED-CT. Each source term was associated with an OMOP CDM *concept_id* identifier which in turn was associated with a SNOMED-CT term (Supplementary Table S3). The OMOP CDM system table source_to_concept_map contains information on mappings between source concept_id and target concept_id. We followed a similar methodology for mapping the units of measurements recorded in CPRD to the Unified Code For Units (UCUM) vocabulary.^37^

We mapped additional structured data fields in CPRD which were recorded using a bespoke lookup system (*entity type* fields which in turn encoded up to eight different types of information) to the LOINC terminology by using an existing mapping previously created by Janssen Pharmaceutical Research & Development, LLC (JNJ_CPRD_ET_LOINC available at https://github.com/OHDSI/ETL-CDMBuilder). Using this mapping, individual fields across *entity types* were mapped to a single LOINC term (and hence a unique *concept_id*) in the CDM. Medications were mapped from Gemscript terms to DM+D and from DM+D to RxNorm.

ETL scripts can be found in reference 38.

### Clinical covariates

Using previously validated EHR phenotyping algorithms [11,20,28], we extracted information from all sources (CPRD, HES, and ONS) on (1) demographics, (2) lifestyle risk factors, (3) clinical measurements and biomarkers (body mass index, total cholesterol, systolic and diastolic blood pressure, platelet count, total white blood cell count, albumin, creatinine, hemoglobin), (4) HF-related comorbidities (atrial fibrillation, chronic obstructive pulmonary disease, acute myocardial infarction, hypertension, cancer (any type)), and (5) related CVD medication (beta-blockers, loop diuretics, ace inhibitors). We translated the existing phenotyping algorithms into the target list of OMOP CDM Concepts using a similar approach described previously.

### Validation and evaluation

Validation of the final transformed data was performed using the Achilles OHDSI tool (https://ohdsi.github.io/Achilles/), which performs about 160 validation checks on the conformance, completeness, and plausibility of the data in the OMOP CDM. Multiple iterations of conversion and validation were performed until all validation checks passed. In addition, we utilized Achilles to create a dashboard of visualizations of key data source characteristics (eg demographics and most occurring clinical events) and inspected them for consistency and clinical plausibility after each transformation iteration and in collaboration with clinical colleagues.

We validated the syntactic mapping by examining the source data and the target tables (and their attributes) for consistency. We validated the semantic mapping by creating, generating, and comparing a set of predefined metrics across both original data and CDM converted data. Specifically, we described and compared the raw and OMOP CDM datasets using descriptive statistics. For clinical measurements, medians of measured values and their standard deviation were compared. For other metrics, the numbers/percentages of patients identified with particular conditions were compared.

## RESULTS

We successfully converted 1 099 195 384 rows of data across three national EHR sources to the OMOP CDM. We mapped 109 772 terms across five controlled clinical terminologies used in the source EHR data for diagnoses, procedures, observations, measurements, deaths, devices, and medication to CDM Concepts (Table 1). Specifically, we mapped 66 245 Read V2 terms, 9187 ICD-10 codes, 495 ICD-9 codes, and 8428 OPCS-4 codes. We observed incomplete mappings in terms related to drug codes where 62.5% of Gemscript codes were mapped successfully to RxNorm (via DM+D).

**Table 1:** Mapping coverage for disease and drug clinical terminologies used across the entire cohort in raw CPRD, HES, and ONS and converted to the OMOP CDM standard dictionary

	Total unique terms in terminology	Total mapped terms (%)	Unique terms used in events	Used mapped terms (%)	Total unique events	Total excluded events (%)	Total mapped events (%)
**Read**	111 163	82.13	67 886	97.58	320 328 788	0.22	97.42
**ICD-9**	6519	99.98	495	100	13 130	0.92	100
**ICD-10**	17 934	85.85	10 158	90.44	31 905 144	0.01	99.09
**OPCS-4**	11 000	99.01	8474	99.45	8 453 813	0	99.88
**Drugs**	66 970	60.09	40 647	62.53	264 589 509	1	92.67
**Units**	287	45.29	22	72.72	27 036	1.55	99.95
**Entity types—laboratory results**	259	51.35	245	54.28	125 581 411	0.59	54.06
**Entity types—test**	324	97.22	324	97.22	151 645 201	12.24	98.16

Using the raw primary care consultations (CPRD) and hospitalization tables (HES), we generated 216 581 914 (211 209 045 for CPRD + 5 372 869 for HES) unique encounter events (stored in the OMOP CDM Visit Occurrence table). Specifically, in CPRD, we created a unique visit occurrence identifier by combining the numeric primary care consultation identifier, the numeric patient identifier, and the date of consultation. In HES, we uniquely identified visits by combining the hospitalization identifier, the numeric patient identifier, and the date of admission to the hospital. A total of 88 988 consultations and 63 hospitalizations were not mapped during the ETL due to missing consultation/admission dates.

We identified 502 723 patients with the incident and prevalent HF recorded across three EHR sources (CPRD, HES, and ONS) in the original data (Table 2). For these patients, we extracted all data across 20 source tables and performed the conversion to the OMOP CDM resulting in a cohort of 502 367 patients. The observation period start date was defined as the greatest of patient registration date or the date a practice started submitting research-quality data (defined as Up To Standard date by the CPRD). We defined the observation period end as the earliest of (1) the date the patient deregistered from the primary care practice, (2) the date the patient’s practice last submitted data, (3) the patient’s death date defined from the national mortality register (ONS), or (4) the study end date (08/03/2016). A number of patients (*n* = 356) were rejected from the ETL pipeline during the conversion to the CDM as the raw data contained inconsistent observation period information (the observation period start date preceded the observation period end date).

**Table 2:** Cohort summary and comparison between the entire cohort of raw CPRD, HES, and ONS data the OMOP CDM cohort

	CPRD-HES-ONS source data	OMOP CDM data
***n***	502 723	502 367
Median follow-up (IQR)	9.56 (10.39)	9.56 (10.39)
Demographics		
Female (%)	52.39	52.4
Caucasian (%)	90.81	90.46
Most deprived fifth (%)	15.18	15.18
Lifestyle		
Smoker (%)	324 755 (64.59)	331 445 (65.97)
Never smoker (%)	155 995 (31.03)	149 569 (29.77)
Clinical measures mean (SD) or median (IQR)		
BMI (kg/m^2^)	28.9 (6.44)	28.9 (6.44)
SBP (mmHg)	143.07 (22.42)	143.07 (22.42)
DBP (mmHg)	80.05 (12.19)	80.05 (12.19)
Platelets	2.39 (3.53)	2.39 (3.53)
Total WBC counts	7.49 (2.88)	7.49 (2.88)
Albumin	40.71 (4.5)	40.71 (4.5)
Creatinine (µmol/L)	102.76 (58.09)	102.76 (58.09)
Hemoglobin	129.92 (18.22)	129.92 (18.22)
Medication		
Loop diuretics (%)	42.2	42.2
ACE-I (%)	50.2	50.1
Βeta-blockers (%)	48.3	48.2

BMI, body mass index; SBP, systolic blood pressure; DBP, diastolic blood pressure; WBC, white blood cell count; ACE-I, angiotensin-converting enzyme (ACE) inhibitors; IQR, interquartile range

We extracted and compared (Tables 2 and 3) information on clinical comorbidities, lifestyle risk factors, and key demographic fields between source and converted data. Specifically, we mapped 2217 Read, ICD-10, ICD-9, and OPCS-4 terms used across existing phenotyping algorithms to 1266 unique OMOP CDM Concepts. We investigated the accuracy of comorbidities’ mappings for each source terminology separately (Supplementary Table S2). We observed the biggest inconsistency in comorbidities definitions encoded by the Read ontology. Inconsistencies were further quantified by examining the unmapped source and incorrectly mapped target encounter events.

**Table 3:** Overall comorbidity comparison between the entire cohort of raw CPRD, HES, and ONS data the OMOP CDM cohort

Comorbidity	Unique patients % (n)		Unmapped patients % (*n*)	Incorrectly mapped patients % (*n*)
	Original	OMOP CDM		
**AF**	35.39 (177 954)	35.40 (177 866)	0.05 (91)	0.001 (3)
**COPD**	49.55 (249 119)	53.33 (267 925)	0.005 (13)	7.02 (18 819)
**T2DM**	23.86 (119 968)	24.09 (121 059)	0.23 (280)	1.13 (1371)
**AMI**	20.29 (102 020)	20.31 (102 028)	0.01 (11)	0.02 (19)
**HT**	65.84 (331 011)	65.86 (330 884)	0.041 (138)	0.003 (11)
**Cancer (all types)**	26.86 (135 047)	27.34 (137 380)	0.048 (65)	1.74 (2398)

AF, atrial fibrillation; COPD, chronic obstructive pulmonary disease; T2DM, type 2 diabetes, AMI, acute myocardial infarction; HT, hypertension.

Data quality assessment based on database profiling summary statistics (generated using Achilles Heel), uncovered four types of data quality and consistency issues: records outside valid observation period in condition (27.8%), visit (12.6%), drug exposure (12.5%), and death records (38.3%); invalid person_id in visit (7%), condition (4.3%), and drug exposure (11.4%) records; invalid visit_id in drug exposure records (19%) and invalid start and end date (end date < start date) in visit records (33 records out of ∼216M).

Records with a date found as to be outside observation periods were fully mapped and could cause potential issues only with tools operating over OMOP CDM, for example, Atlas. Direct validation described in this paper is not affected by these records, which were given by the source data quality and could be reduced by modification of the formula for observation period calculation. All reported *invalid person_id* errors were caused by the records related with 356 unmapped patients. *Invalid visit_id* errors were caused by an inconsistency found in the source data since not all diagnosis/prescription records (in the *therapy/clinical* CPRD tables) had a corresponding record in the primary care consultations table (CPRD *consultation* table). A detailed overview of the most frequently utilized and mapped terms from the source vocabularies is provided in Supplementary Tables 4, 5, and 6.

## DISCUSSION

In this study, we extracted and mapped a cohort of 502 723 HF patients derived from three national EHR data sources spanning primary care, hospitalizations, and mortality into the OMOP CDM. We performed syntactic and semantic validation of the resulting data set by repeating a series of descriptive analyses in both raw and converted datasets and analyzed similarities and differences between data sets.

Diagnostic and procedure codes from the Read, ICD-10, ICD-9, and OPCS-4 terminologies were mapped with a coverage between 82% and 99% (Table 1). The impact of mapping coverage on the total number of mapped events was minimal as the percentage of mapped events was between 97.4% and 100%. In line with previous research, primary care prescriptions in the CPRD which are recorded using a bespoke vocabulary were challenging to map to CDM Concepts with only 60% mapped. Despite this however, ∼93% of all prescription records in the source data were successfully mapped which indicates that the drugs which were not mapped to Concepts are very infrequently used.

The main reason leading to a reduced proportion of mapped medications prescribed in the source data was the lack of equivalent concepts, that is, the source concept is either too specific or too general to be directly mapped to a destination concept. In Supplementary Table S7, we provide a breakdown of the top 10 (based on frequency) unmapped drugs all of which do not have a direct, valid mapping to RxNorm Extension. For example, “aqueous cream” is used as an active substance to multiple products but does not exist as a standalone product and similarly, Gaviscon products exist only for specific volumes and concentrations. This is likely due to the fact that 80% of the medications prescribed in UK primary care are nonproprietary (generic) versions rather than the commercial branded equivalents. A potential solution to address this challenge would potentially involve creating bespoke mappings linking the prescription’s active substance to a more generic target concept. This would be a manual process with significant resource requirements and as such as not attempted at this stage given the fact that the current mapping covered 93% of prescription records and the low impact of omitting the unmapped prescriptions from our study.

Structured data fields (entity types) capturing clinical examination findings (derived from the CPRD *additional* table) and laboratory and other miscellaneous test results (derived from the CPRD *test* table) were the most challenging to map. Specifically, only 54% of measurement-related terms and events were successfully mapped mainly due to two reasons. Firstly, the mapping transformed the original wide format to a long format. An entity type can have up to eight distinct structured data fields attached to it—for example, the entity type for blood pressure has eight data fields: diastolic blood pressure, systolic blood pressure, korotkoff sounds, time of measurement, laterality, patient posture, and cuff position. The majority of these however do not contain any data (as they are not systematically captured by the primary care physician). For example, the time of the blood pressure measurement is only defined in 4.7% of the rows in the source data (Supplementary Table S8). Similarly, for laboratory tests, entity type 467 “Procedures, specimens and samples” accounts for approximately 55% of unmapped rows but the majority of records associated with it contain “0” as a value which is used to denote “value not entered.” As a result, during this wide-to-long conversion, the number of unmapped events generated during the conversion ETL is artificially inflated given that these fields are missing in the raw data. Secondly, not all concepts captured by the structured data fields had an equivalent mapping in LOINC. For example, LOINC does not seem to have an equivalent concept for Korotkoff Sounds nor concepts that adequately capture administrative tasks around repeat prescriptions such as the date of the next review or the staff member due to review these medications. Similar to the way medications were mapped while we did not add bespoke mappings further examination could potentially increase coverage. Despite these reasons, we did not observe a negative impact on the precision of the standard tests and measurements we mapped for our study (Table 2).

For both medications and clinical findings/laboratory results, creating bespoke mappings in collaboration with clinicians and doing further ETL iterations could potentially lead to higher mapping coverage percentages. With regards to medications, mapping based on the active substance rather than the product name could potentially alleviate some of the mismatches between UK and US pharmaceutical products that caused some mappings to fail. For clinical examination findings and laboratory tests, the issue is more complex as a large percentage of the unmapped records is due to missing data rather than failed mappings. Prioritizing specific entity types based on study requirements combined with missingness could lead to a more manageable list of fields that require bespoke mappings. In both cases however, further iterations would require significantly more human and computational resources.

In total, there were 1165 ICD-10 codes which were not mapped. The majority of unmapped ICD-10 codes were from the W00-X59 “Other External Causes of Accidental Injury” chapter—specifically, 502 codes were from the “W00-W19 Falls” group and 466 codes spanned both W and X groups, for example codes from groups X61 and X60 associated with intentional self-harm. Supplementary Table S10 provides the top ten most frequent unmapped ICD-10 codes and the overall percentage of unmapped events that are associated with each code. Interestingly, these top ten codes all do not exist in the ICD-10 vocabulary. The first three characters are valid ICD-10 codes, but the last digit is not. For example, while “W19” exists in the ICD-10 version that the UK utilizes, W19.9 does not and therefore a mapping to a standard concept was not available. An alternative mapping strategy would be to map these codes first to their category (eg W19.0 to W19) and map that to a standard OMOP concept. However, this was not performed at this stage given that it could potentially lead to information loss by mapping a specific term to a broader parent term. Also the overall prevalence of these codes in the raw data is low and the impact of not including these in the study was assessed to be minimal as neither chapters are related to HF—two codes (W19.0 Unspecified fall home while engaged in sports activity and W19.9 Unspecified fall home during unspecified activity) accounted for approximately 50% of unmapped events and related to unspecified falls at home.

We observed good overall consistency when comparing key demographic, lifestyle risk factors, and related clinical comorbidities between the original raw data from three EHR sources and the CDM data. Despite the worst vocabulary coverage, the best results were observed in clinical measurements as the mapping was binary. Inconsistency in demographics details was observed in the patient’s ethnicity classification which is recorded in two separate source data (CPRD and HES). However, only HES ethnicity records were used inside the ETL process, which caused a difference between the source and target in Caucasians (∼0.35%).

The largest discrepancies were comorbidities were found in COPD, cancer status, and smoking information but the difference for these remained below 4% between the source and target events. Differences were mainly caused by incorrect mappings which in term translated to broader disease phenotypes. For example, in COPD, multiple Read codes used in CPRD were translated to the same Concept including terms which were not originally presented in the phenotyping algorithm (Supplementary Figure S1).

In this work, we chose HF as an exemplar as it showcases the strengths of national linked electronic health records but the findings we present here are generalizable to other conditions defined in similar data. For example, the HF population we defined includes information on related comorbidities (eg cancer), biomarkers (eg blood pressure, white blood cells), medications (eg beta-blockers) which can be used as a guide for researchers to define comparable clinical populations. HF as a disease itself is defined by diagnoses across multiple sources (eg primary care, hospitalizations, and mortality) and the approach illustrated here can serve as a template for defining other diseases in a similar manner. Other diseases however might require some manual mappings to be created, for example to extract specific biomarker measurements or medications which were not mapped in our ETL workflow but that should be relatively straightforward to do.

## CONCLUSIONS

In this study, we demonstrated how the OMOP CDM can be successfully used to implement existing EHR phenotyping algorithms using multiple EHR sources to the CDM. Our results showed that translated phenotyping algorithms and the population derived from the displayed similar results between the source and CDM datasets. Widespread usage of the CDM, especially in commonly used datasets, can enable reproducible research at scale at lower costs.

## SUPPLEMENTARY MATERIAL

Supplementary material is available at *Journal of the American Medical Informatics Association* online.

## Contributors

S.D. conceived and designed the study. V.P., M.M., and S.P. implemented the ETL pipeline and conversion to OMOP CDM. V.P. analyzed the data. S.D. and V.P. wrote the manuscript. R.D., F.A., H.H., and R.T.L. made substantial revisions to the manuscript. All authors reviewed and interpreted the results, commented on the report, contributed to revisions, and read and approved the final version.

## Supplementary Material

ooab001_Supplementary_Data
