# Supplementary material for: Transforming and evaluating electronic health record disease phenotyping algorithms using the OMOP common data model: a case study in heart failure
Source: JAMIA Open. 2021 Feb 4;4(3):ooab001. doi: 10.1093/jamiaopen/ooab001 (PMC8423424; doi:10.1093/jamiaopen/ooab001)
Supplement: ooab001_Supplementary_Data [file ooab001_supplementary_data.docx]

**Supplementary tables and figures**


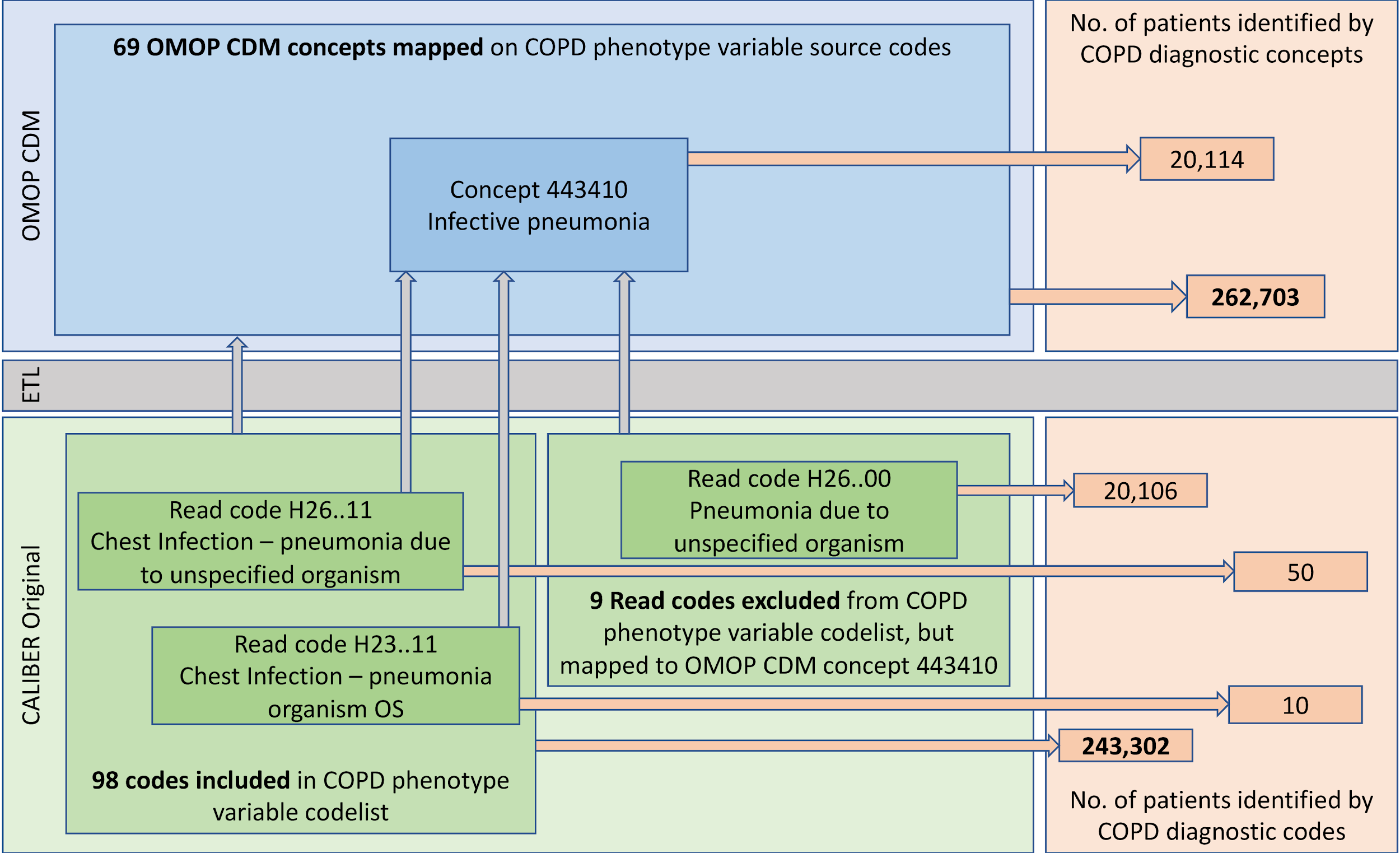


**Supplementary Figure 1**: Example of inconsistency between original and converted records demonstrated using the Chronic Obstructive Pulmonary Disease (COPD) phenotype. Multiple source terminology terms codes (Read codes in green boxes) are mapped onto the same OMOP CDM target concept (blue box). The mapped concept however includes a broader set of clinical diagnoses which are not part of the original COPD phenotype. As a result, the number of patients retrieved (orange boxes) in the raw data using the original phenotype terms (243,302) is significantly lower than the number of patients retrieved using the OMOP CDM phenotype (262,703). The main culprit of this inconsistency is the Main result difference is caused by the Read code *H26..00 Pneumonia due to unspecified organism* used in more that 20,106 patients, which is excluded from COPD phenotype, but mapped to the same concept of *Infective pneumonia* as other Read codes from the phenotype.

COPD: Chronic Obstructive Pulmonary Disease, CDM: Common Data Model; OS: Otherwise Specified; OMOP: Observational Health Data Science and Informatics

**Supplementary Table 1**: Comparison of code mappings between source data across three electronic health record sources and target OMOP CDM data

CPRD: Clinical Practice Research Datalink; HES: Hospital Episode Statistics; ONS: Office for National Statistics; OPCS4: OPCS Classification of Interventions and Procedures version 4; CDM: Common Data Model; AF: Atrial Fibrillation; COPD: Chronic Obstructive Pulmonary Disease; T2DM: Type 2 Diabetes; AMI: Acute Myocardial Infarction; HT: Hypertension; OMOP: Observational Health Data Science and Informatics

| **Comorbidity** | **Source codes (n)** | | | | | **Target concepts (n)** |
| --- | --- | --- | --- | --- | --- | --- |
|  | **CPRD** | **HES** | **OPCS** | **ONS** | **Total** |  |
| **AF** | 23 | 7 | 10 | - | 40 | 31 |
| **COPD** | 98 | 18 | - | 16 | 132 | 73 |
| **T2DM** | 455 | 47 | - | - | 502 | 300 |
| **AMI** | 68 | 18 | - | 4 | 90 | 57 |
| **HT** | 103 | 14 | - | - | 117 | 80 |
| **Cancer (all types)** | 649 | 434 | - | 253 | 1336 | 725 |

**Supplementary Table 2**: Comparison of comorbidities between source data across three electronic health record sources and target OMOP CDM data.

CPRD: Clinical Practice Research Datalink; HES: Hospital Episode Statistics; ONS: Office for National Statistics; OPCS4: OPCS Classification of Interventions and Procedures version 4; CDM: Common Data Model; AF: Atrial Fibrillation; COPD: Chronic Obstructive Pulmonary Disease; T2DM: Type 2 Diabetes; AMI: Acute Myocardial Infarction; HT: Hypertension; OMOP: Observational Health Data Science and Informatics

| **Comorbidity** | **CPRD % (n)** | | **HES % (n)** | | **OPCS % (n)** | | **ONS % (n)** | |
| --- | --- | --- | --- | --- | --- | --- | --- | --- |
|  | original | OMOP CDM | original | OMOP CDM | original | OMOP CDM | original | OMOP CDM |
| **AF** | 20.36 (102,366) | 20.35 (102,232) | 32.88 (165,297) | 32.9 (165,294) | 4.42 (22,228) | 4.42 (22,228) | - | - |
| **COPD** | 48.4 (243,302) | 52.29 (262,703) | 4.24 (21,308) | 4.24 (21,308) | - | - | 17.15 (86,200) | 17.16 (86,200) |
| **T2DM** | 20.35 (102,299) | 20.58 (103,406) | 19.63 (98,668) | 19.64 (98,669) | - | - | - | - |
| **AMI** | 13.97 (70,247) | 13.99 (70,292) | 13.91 (69,919) | 13.92 (69,919) | - | - | 3.3 (16,599) | 3.3 (16,566) |
| **HT** | 51.28 (257,813) | 51.26 (257,496) | 56.52 (284,161) | 56.56 (284,160) | - | - | - | - |
| **Cancer (all types)** | 18.21 (91,527) | 18.54 (93,145) | 20.23 (101,700) | 20.5 (103,007) | - | - | 7.39 (37,164) | 7.45 (37,426) |

**Supplementary Table 3**: Examples of mappings of different concepts: primary care diagnoses, secondary care diagnoses, units, secondary care procedures, primary care continuous measurements and primary care prescriptions.

mmol/L: millimol per litre; CPRD: Clinical Practice Research Datalink; OPCS4: OPCS Classification of Interventions and Procedures version 4; UCUM: Unified Code for Units of Measure (UCUM); 10th revision of the International Statistical Classification of Diseases and Related Health Problems; DM+D: Dictionary of medicines and devices; BP Blood Pressure; OMOP: Observational Health Data Science and Informatics

| **Source Term** | **Source terminology** | **Source Concept ID** | **Mapping** | **Target Concept ID** | **Target** | **Target Term** |
| --- | --- | --- | --- | --- | --- | --- |
|  | **Source code** |  |  |  | **Target code** |  |
| **Type 1 diabetes mellitus** | Read | 45420112 | direct | 201254 | SNOMED CT | Type 1 diabetes mellitus |
|  | C108.12 |  |  |  | 46635009 |  |
| **Dysthymia** | ICD10 | 45586238 | direct | 433440 | SNOMED CT | Dysthymia |
|  | F34.1 |  |  |  | 78667006 |  |
| **mmol/L** | CPRD Unit | 2000068400 | direct | 8753 | UCUM | millimole per liter |
|  | 96 |  |  |  | mmol/L |  |
| **Partial splenectomy** | OPCS4 | 44511356 | direct | 4281839 | SNOMED CT | Partial splenectomy |
|  | J70.1 |  |  |  | 67097003 |  |
| **Examination findings - Blood pressure** | CPRD Entity | 2000068426, 2000068406,... | JNJ_CPRD_ET_LOINC | 3004249, 3012888,... | LOINC | Systolic BP, Diastolic BP, ... |
|  | 1 (Diastolic BP, Systolic BP,...) |  |  |  | 8480-6, 8462-4, .. |  |
| **Simvastatin 10mg tablets** | CPRD product code | 2000035557 | gemscript, DM+D | 1539463 | RxNorm | Simvastatin 10 MG Oral Tablet |
|  | 42 |  |  |  | 314231 |  |

**Supplementary Table 4**: Top five diagnoses and observation with mapped concept descriptions in CALIBER OMOP CDM across the entire heart failure cohort.

O/E: On Examination; BP: Blood pressure; OMOP: Observational Health Data Science and Informatics

| **Source vocabulary (Read/ICD-10)** | **Source code and term** | **Mapped SNOMED-CT** | **% of rows in OMOP CDM** |
| --- | --- | --- | --- |
| Read | 246..00 - O/E - blood pressure reading | 163020007 - O/E - blood pressure reading | 13.32 |
| ICD10 | I10 - Essential (primary) hypertension | 59621000 - Essential hypertension | 2.13 |
| Read | 246..11 - O/E - BP reading | 163020007 - O/E - blood pressure reading | 1.63 |
| Read | 242..00 - O/E - pulse rate | 162986007 - O/E - pulse rate | 1.28 |
| ICD10 | I48 - Atrial fibrillation and flutter | 17366009 - Atrial arrhythmia | 1.08 |

**Supplementary Table 5**: Top administrative procedures with mapped concept descriptions in CALIBER OMOP CDM across the entire heart failure cohort.

| **Source vocabulary (Read)** | **Source code and term** | **Mapped SNOMED-CT** | **% of rows in OMOP CDM** |
| --- | --- | --- | --- |
| Read | 6A...00 - Patient reviewed | 171430003 - Patient reviewed | 7.46 |
| Read | 9Z...00 - Administration NOS | 416118004 - Administration | 3.91 |
| Read | 8B3S.00 - Medication review | 182836005 - Review of medication | 2.78 |
| Read | 8B31400 - Medication review | 182836005 - Review of medication | 2.54 |
| Read | 81H..0 - Dressing of wound | 182531007 - Dressing of wound | 2.42 |

**Supplementary Table 6**: Top five drugs with mapped concept descriptions in CALIBER OMOP CDM across the entire heart failure cohort.

| **Source vocabulary (CPRD PRODUCT)** | **Source code and term** | **Mapped RxNorm** | **% of rows in OMOP CDM** |
| --- | --- | --- | --- |
| CPRD PRODUCT | 3 - Aspirin 75mg dispersible tablets | 104475 - Aspirin 75 MG Disintegrating Oral Tablet | 3.41 |
| CPRD PRODUCT | 6 - Furosemide 40mg tablets | 313988 - Furosemide 40 MG Oral Tablet | 2.21 |
| CPRD PRODUCT | 2 - Bendroflumethiazide 2.5mg tablets | 308614 - Bendroflumethiazide 2.5 MG Oral Tablet | 2.04 |
| CPRD PRODUCT | 51 - Simvastatin 40mg tablets | 198211 - Simvastatin 40 MG Oral Tablet | 1.73 |
| CPRD PRODUCT | 7 - Paracetamol 500mg tablets | 198440 - Acetaminophen 500 MG Oral Tablet | 1.68 |

**Supplementary Table 7**: The ten most frequently-prescribed medications in Clinical Practice Research Datalink (CPRD) that were not mapped to OMOP CDM Concepts. For a given prescription product, the table provides the number of unique patients with at least one prescription during the observation period, the number of prescription events and the proportion of overall unmapped events (n=19,184,577) that this prescription accounts for.

| **CPRD product code** | **Term** | **N patients** | **N events** | **% events** |
| --- | --- | --- | --- | --- |
| 14 | Gaviscon Oral solution (Reckitt Benckiser Healthcare (UK) Ltd) | 66,158 | 837,592 | 4.3659 |
| 29 | Amlodipine besilate 5mg tablets | 47,993 | 772,259 | 4.0254 |
| 12 | Lactulose 3.35g/5ml Oral solution | 70,702 | 738,898 | 3.8515 |
| 59 | Aqueous cream | 78,617 | 452,022 | 2.3561 |
| 71 | Amlodipine besilate 10mg tablets | 22,541 | 438,824 | 2.2873 |
| 54 | Fybogel sugar free Granules (Reckitt Benckiser Healthcare (UK) Ltd) | 43,853 | 424,829 | 2.2144 |
| 729 | Amlodipine maleate 5mg tablets | 25,014 | 411,117 | 2.1429 |
| 4611 | Gaviscon advance Oral suspension (Reckitt Benckiser Healthcare (UK) Ltd) | 31,419 | 299,867 | 1.563 |
| 898 | Ventolin evohaler 100 100microgram/inhalation Pressurised inhalation (Glaxo Wellcome UK Ltd) | 26,250 | 294,036 | 1.5326 |
| 1262 | Nifedipine 12 20mg Modified-release tablet | 9,255 | 283,265 | 1.4765 |

**Supplementary Table 8**: The ten most frequently-used structured data (entity type) fields in the Clinical Practice Research Datalink (CPRD) *additional* table used to record clinical examination findings. The table provides a breakdown by entity type and data field of the number of unique patients and events where data were defined and the proportion of unmapped records this entry accounts for.

| **Term (entity type - data field)** | **N patients** | **N patients with data** | **N events (% of total unmapped events)** | **N events with data (%)** |
| --- | --- | --- | --- | --- |
| Examination Findings Blood pressure Event Time (1-4) | 487,416 | 142,822 | 12,474,116 (21.5%) | 591,484  (4.74%) |
| Examination Findings Blood pressure Korotkoff (1-3) | 487,416 | 46,748 | 12,474,116  (21.5%) | 200,944  (1.61) |
| Lifestyle Smoking Cigars per day (4-3) | 480,859 | 6,314 | 3,887,228  (6.7%) | 13,613  (0.35) |
| Lifestyle Smoking Ounces of tobacco (4-4) | 480,859 | 7,510 | 3,887,228  (6.7%) | 13,124  (0.34) |
| Miscellaneous Repeat Medication Review Due date (461-1) | 232,935 | 232,934 | 2,611,669  (4.5%) | 2,609,143  (99.9) |
| Miscellaneous Repeat Medication Review Seen by (461-2) | 232,935 | 232,935 | 2,611,669  (4.5%) | 2,611,669  (100%) |
| Miscellaneous Repeat Medication Review Review date (461-3) | 232,935 | 229,988 | 2,611,669  (4.5%) | 2,135,592 (81.77%) |
| Miscellaneous Repeat Medication Review Next review date (461-4) | 232,935 | 229,799 | 2,611,669  (4.5%) | 2,076,112 (17.49%) |
| Lifestyle Alcohol Stop Date (5-4) | 437,699 | 5,316 | 1,941,719  (3.34%) | 6,317  (0.33%) |
| Lifestyle Advice given Format of advice Advice Given (23-1) | 299,804 | 299,804 | 1,529,054  (2.63%) | 1,529,054  (100%) |

**Supplementary Table 9**: The five most frequently-used structured data (entity type) fields in the Clinical Practice Research Datalink (CPRD) *test* table used to record laboratory test results and other miscellaneous findings. The table provides a breakdown by entity type and data field of the number of unique patients and events where data were defined and the proportion of unmapped records this entry accounts for.

| **Term (entity type - data field)** | **N patients** | **N patients with data** | **N events (% of total unmapped events)** | **N events with data (%)** |
| --- | --- | --- | --- | --- |
| Procedures, specimens and samples (467) | 22,205 | 22,205 | 40,366  (54.98%) | 40,366  (100%) |
| Immunology screening tests (472) | 15,398 | 412 | 27,263  (31.05%) | 528  (1.94%) |
| Alpha fetoprotein weeks  (154-8) | 6,280 | 1 | 9,553  (10.88%) | 1  (0.01%) |
| Other Lab Result Information (480) | 884 | 325 | 7,903  (9%) | 2,012  (25.46%) |
| Lung function pre-steroids (486) | 992 | 829 | 1,324  (1.5%) | 1,002  (75.68%) |

**Supplementary Table 10**: Top 10 unmapped ICD-10 codes and proportion of their occurrence.

| **ICD-10 code** | **N patients** | **N events** | **% of unmapped events** |
| --- | --- | --- | --- |
| W19.0 Unspecified fall home while engaged in sports activity | 29,642 | 73,569 | 25.5679 |
| W19.9 Unspecified fall home during unspecified activity | 26,699 | 69,678 | 24.2157 |
| W01.0 Fall on same level from slipping | 19,220 | 44,029 | 15.3017 |
| W18.0 Other fall on same level home while engaged in sports activity | 13,302 | 30,251 | 10.5133 |
| W19.1 Unspecified fall home while engaged in leisure activity | 8,257 | 19,474 | 6.7679 |
| W19.2 Unspecified fall home while working for income | 6,906 | 15,048 | 5.2297 |
| W10.0 Fall (on)(from) escalator | 6,840 | 14,728 | 5.1185 |
| W06.0 Fall from bed | 5,643 | 12,239 | 4.2535 |
| W01.9 Fall on same level from slipping tripping and stumbling home during unspecified activity | 5,245 | 11,204 | 3.8938 |
| W01.4 Fall on same level from slipping tripping and stumbling home while resting sleeping eating | 4,461 | 9,364 | 3.2543 |
